# Phenotypic spectrum and hormonal profile in hypogonadotropic hypogonadism

**Published:** 2014-03-25

**Authors:** D Păun, I Gherlan, I Popescu, C Procopiuc, C Dumitrescu, A Brehar, D Dinu, C Neamtu, C Poiana, C Dumitrache

**Affiliations:** * ”C.I. Parhon” National Institutue of Endocrinology, Bucharest, “Carol Davila” University of Medicine and Pharmacy, Bucharest

**Keywords:** Hypogonadotropic hypogonadism, morphotypes, hormonal profile

## Abstract

Abstract

Background: Hypogonadotropic hypogonadism (HH) is characterized by inappropriately low serum concentration of LH (luteinizing hormone) and FSH (follicle-stimulated hormone) in the setting of hypogonadism. A number of pathologic processes cause Hypogonadotropic hypogonadism but it can also occur as a part of various congenital syndromes.

Objectives. To characterize the morphotypes and the hormonal profile of the HH patients enrolled in the COST Action BM1105 within “C.I. Parhon” National Institute of Endocrinology from May 2012 onward.

Methods. The eligible patients were selected by using a general protocol that included: a detailed familial and personal history; a clinical evaluation focusing on genital development; a hormonal evaluation that aimed to exclude the acquired causes of HH and to characterize the basal/stimulated (triptoreline) profile of gonadotropins; a DNA extraction for genetic studies.

Results: We examinated the medical records of patients admitted in our institute with the diagnosis of hypogonadotropic hypogonadism from May 2012 onward. There were 19 patients: 12 males and 7 females, age at diagnosis 28.03±11.45 years (13.4-56 years). The phenotypic expressions were variable and the hormonal evaluation showed low values of basal and stimulated gonadotropins.

Conclusions: Although hypogonadotropic hypogonadism is a rare disease, the monospeciality profile of National Institute of Endocrinology enable the enrolment of a high number of patients in order to create clinical guidelines for evaluation/diagnosis and for treating GnRH deficient patients.

## Introduction

Episodic stimulation of LH and FSH secretion from the pituitary by gonadotropin releasing hormone (GnRH) represents the initial neuroendocrine step in the reproductive cascade. GnRH command pulsatile gonadotropin secretion, modulate gonadal steroid feedback and determine the initiation of pubertal development and fertility across the life [**1**].

Isolated GnRH deficiency (IGD) is caused by impaired gonadotropin release in the setting of otherwise normal anterior pituitary anatomy and function and in the absence of secondary causes of hypogonadotropic hypogonadism. Individuals with IGD have normal pituitary function tests and their hypogonadism typically responds to a physiologic regimen of exogenous GnRH [**2**].

 GnRH deficiency is caused by complete or partial absence of GnRH-mediated release of LH and FSH. In addition to the reproductive defect, approximately two thirds of individuals with IGD have and impaired sense of smell (anosmia/hyposmia) [**3**]. In the presence of anosmia, isolated GnRH deficiency is called Kallmann syndrome and in the presence of a normal smell it is called normosmic IGD.

 The signs of gonadotropin deficiency in a male may be present at birth but typically the significance of these findings is not recognized until puberty. Cryptochidism and micropenis can be a manifestation of an early impairment in the reproductive axis in boys, especially when associated with abnormally low serum concentrations of gonadotropins and testosterone in the first month of life [**4**]. The rate of linear growth is usually normal but most individuals have a eunuchoid body habitus.

 At puberty, most individuals with IGD have abnormal sexual maturation, usually with incomplete development of secondary sexual characteristics. In adult male, the impaired sexual development means prepubertal testicular volume, absence of secondary sexual features (facial and axillary hair growth and deepening of the voice and decreased muscle mass). Females have little or no breast development and primary amenorrhea. Pubic hair growth is normal in both sexes because adrenal maturation proceeds normally.

 Isolated GnRH deficiency is characterized by low or normal serum concentration of LH and FSH in the setting of low circulating concentrations of sex steroids (testosterone and estradiol). LH secretions are apulsatile and responsiveness to a regimen of physiologic doses of exogenous GnRH [**5**].

## Objectives

To characterize the morphotypes and the hormonal profile of the patients with hypogonadotropic hypogonadism within “C.I. Parhon” National Institute of Endocrinology enrolled in the COST Action BM 1105 from May 2012 onward.

## Materials and Methods

The eligible patients were selected using a general protocol that included:

 A. Medical History

 - Family medical history questionnaire:

Pattern of growth and age of pubertal onset of parents

 History of: anosmia, deafness, craniofacial defects, micropenis/cryptorchidism, renal agenesis, early-onset obesity, primary adrenal failure

 - Sexological medical history

 - Personal medical history questionnaire:

Micropenis/cryptorchidism

Craniofacial defects

 Anosmia, deafness, abnormal vision

Renal agenesis

Mental retardation

Multiple adrenal crisis in boys

Chronic disease (including HIV infection), eating disorders

Performance sport

Medication: glucocorticoids, antiphychotics, chemo/radiotherapy

Early onset obesity

Growth chart

B. Physical examination

- Height, weight, BMI, skeleton ratios: sitting/total height and arm span/height

- Craniofacial and skeletal dysmorphism

- Ichthyosis

- Thyroid and adrenal status

- Cardiologic examination for heart malformations

- Neurologic evaluation: synkinesis, nystagmus, paraplegia, mental retard, high intracranial pressure

- Males: micropenis/ cryptorchidism, testicular volume (Tanner stage G), facial and corporal pilosity, gynecomastia

- Females: breast development (Tanner stage B), galactorrhoea, external genitalia appearance 

- Males and females: axilo-pubic pilosity (Tanner stage P), fat distribution

C. Paraclinical evaluation

- Hand and wrist radiography for bone age in children and teenagers (Greulich and Pyle)

- Hemogram

- Complete biochemical evaluation including ferritin level for adult patients with newly onset hypogonadotropic hypogonadism

- Gonadal axis: FSH, LH, estradiol/testosterone (sensitivity: E2 – 5 pg/ml, LH- 0,2 UI/l, FSH-0,2 UI/l)

- Prolactin level

- Adrenal axis: ACTH, cortisol, free urinary cortisol, dexamethasone suppresion test

- Thyroid axis: TSH, FT4

- Inhibin B

- Imaging: MNR, CT, testicular ultrasound in males and pelvic echography for females patients

- DNA extraction for genetic studies 

- GnRH stimulation test used to: distinguish hypogonadotropic hypogonadism from constitutional delay of growth and puberty in teenage patients and exclude isolated FSH or LH deficiency in adult patients.

Triptorelinum was administrated subcutanously 100 microg/mp body surface. FSH, LH was sampled bassaly and after 4 hours while estradiol/testosterone were sampled after 24 hours (**Table 1**).

**Table 1:**
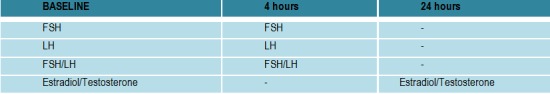
Triptorelinum test protocol

## Results

We examinated medical records of 19 patients admitted in our institute with the diagnosis of hypogonadotropic hypogonadism from May 2012 onward.

There were 19 patients with HH enrolled between May 2012 and August 2013–12 males and 7 females, aged at diagnosis 28.03±±11.45 years (13.4-56 years, 14 adults and 5 adolescents).

**Fig. 1:**
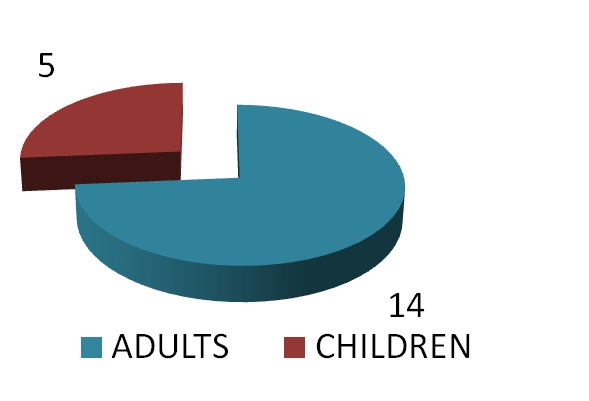
The distribution of the patients according to age

**Fig. 2:**
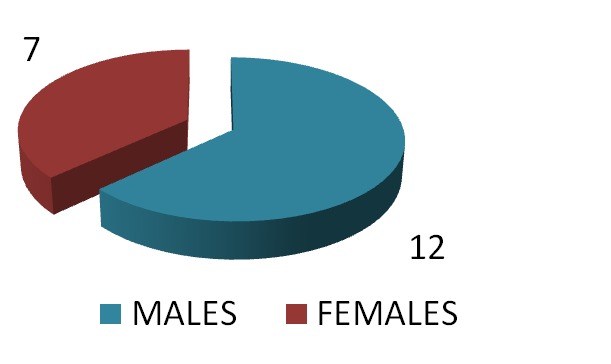
The distribution of the patients according to gender

Family and medical personal history are showed in (**Table 2**).

**Table 2:**
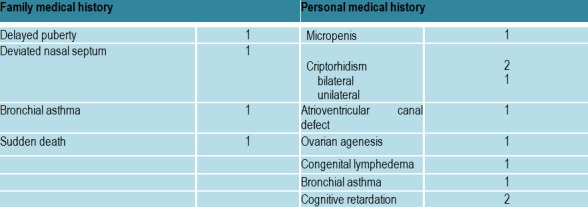
Medical history in patients with hypogonadotropic hypogonadism

The phenotypic expressions were variable (**Table 3**); for example one patient had hyposmia, congenital lymphedema and high arched palate, two patients with normal sense of smell had mental retardation, one patient associated absent lateral superior incisives, absence of septum pellucidum and Verga Ventricle persistence; one female patient had atrioventricular canal persistence.

**Table 3:**
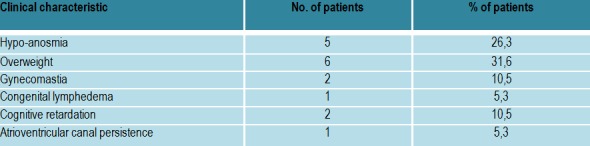
Clinical characteristics in patients with hypogonadotropic hypogonadism

**Fig. 3:**
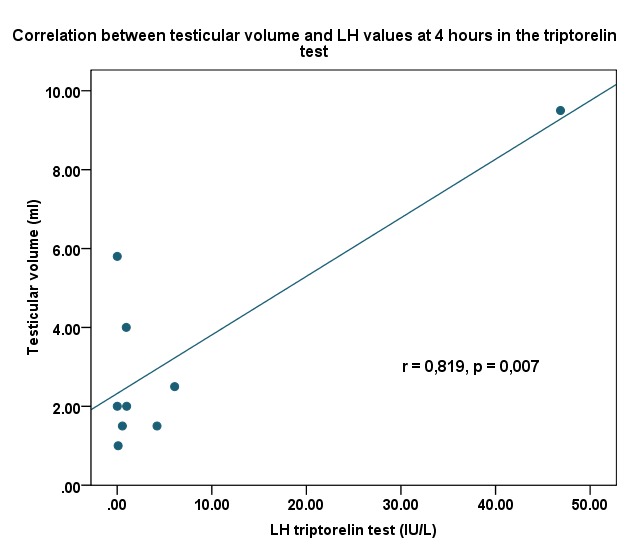
Correlation between testicular volume and LH values in the Triptorelinum test

Paraclinical characteristics (**Table 4**,**Table 5**, **Fig. 3**)

 Evaluation of FSH/LH ratio demonstrated no significant difference between basal and stimulated states, excluding isolated FSH or LH deficiency.

 A cut-off value of stimulated LH≥5 UI/l and a cut-off value of stimulated testosterone ≥0,9 ng/dl /estradiol ≥70 pg/ml were considered for excluding constitutional delay of growth and puberty.

 Adrenal and thyroid axis was found normal in all cases.

**Table 4:**
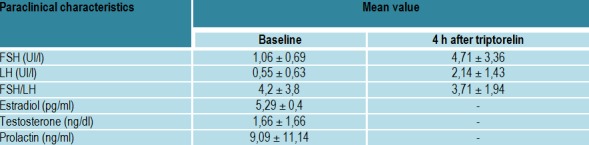
The hormonal evaluation in adults with hypogonadotropic hypogonadism

**Table 5:**
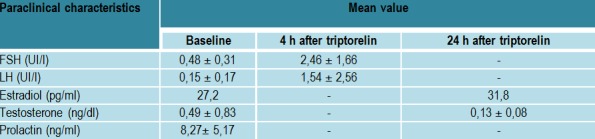
The hormonal evaluation in pediatric patients

## Conclusions

Although hypogonadotropic hypogonadism is a rare disease, the monospeciality profile of the National Institute of Endocrinology enable the enrolment of a high number of patients, the hormonal evaluation of this disease being continuously ongoing. In this way, Romanian endocrinologists may have a leading role in create clinical guidelines for evaluation, diagnosis and for treating GnRH deficient patients and to translate scientific understanding into improved patient care.
